# Supplementary material for: Ownership structure and financial performance: Evidence from Kenyan commercial banks
Source: PLoS One. 2022 May 20;17(5):e0268301. doi: 10.1371/journal.pone.0268301 (PMC9122193; doi:10.1371/journal.pone.0268301)
Supplement: S1 File — (PDF) [file pone.0268301.s001.pdf]

	BANK	YEAR	SO	MO	IO	FO	NIM	EPS	ROA	ROE	TOTAL ASSETS	NPL	LOANS & ADVANCES	BS	CR
1	ABC BANK	2009	0.00%	25.00%	18.30%	14.20%	0.070113	3.093963	0.029069	0.224454	8841000	40801	3992000	6.946501	0.010221
1	ABC BANK	2010	0.00%	25.00%	18.30%	14.20%	0.067257	4.12	0.033237	0.209882	10296561	38744	5288180	7.012692	0.007327
1	ABC BANK	2011	0.00%	25.00%	18.30%	14.20%	0.060176	3.53	0.029855	0.219373	12506895	73511	7073553	7.09715	0.010392
1	ABC BANK	2012	0.00%	33.50%	18.30%	14.20%	0.045898	4.04	0.022224	0.200716	19070779	204767	9789658	7.280368	0.020917
1	ABC BANK	2013	0.00%	33.50%	18.30%	15.60%	0.063595	4.04	0.022514	0.1686	19639370	624165	10851417	7.293128	0.057519
1	ABC BANK	2014	0.00%	33.50%	26.70%	15.60%	0.064363	2.46	0.012564	0.10994	21438729	885124	13127628	7.331199	0.067425
1	ABC BANK	2015	0.00%	33.50%	26.70%	15.60%	0.054505	2.54	0.012092	0.094027	22058297	1967089	14828522	7.343572	0.132656
1	ABC BANK	2016	0.00%	43.00%	26.70%	15.60%	0.044261	1.5	0.007014	0.052477	22422351	2047032	14228599	7.350681	0.143867
1	ABC BANK	2017	0.00%	43.00%	33.30%	16.67%	0.043531	1.31	0.005527	0.043381	24804407	3534602	15382987	7.394529	0.229773
1	ABC BANK	2018	0.00%	43.00%	33.30%	16.67%	0.043282	0.6	0.002308	0.017656	27212707	4232101	17248429	7.434772	0.245362
1	ABC BANK	2019	0.00%	43.00%	33.30%	16.67%	0.041529	1.14	0.004166	0.032388	28680487	3556700	18723123	7.457587	0.189963
1	ABC BANK	2020	0.00%	66.70%	33.30%	16.67%	0.038175	1.25	0.004031	0.034478	32643367	3424863	20977479	7.513795	0.163264
2	BANK OF AFRICA	2009	0.00%	0.00%	100.00%	100.00%	0.031527	1.1	0.011377	0.065339	16915292	145459	9120438	7.228279	0.015949
2	BANK OF AFRICA	2010	0.00%	0.00%	100.00%	100.00%	0.033283	0.16	0.013306	0.141488	26699124	257806	14122485	7.426497	0.018255
2	BANK OF AFRICA	2011	0.00%	0.00%	100.00%	100.00%	0.035391	0.13	0.011172	0.092621	38734220	317730	21639691	7.588095	0.014683
2	BANK OF AFRICA	2012	0.00%	0.00%	100.00%	100.00%	0.03351	0.14	0.009677	0.094564	48957925	553573	29882472	7.689823	0.018525
2	BANK OF AFRICA	2013	0.00%	0.00%	100.00%	100.00%	0.040522	0.18	0.014344	0.115563	52683299	1088758	31091347	7.721673	0.035018
2	BANK OF AFRICA	2014	0.00%	0.00%	100.00%	100.00%	0.037054	0.03	0.002316	0.018211	62211641	1639985	38463876	7.793872	0.042637
2	BANK OF AFRICA	2015	0.00%	0.00%	100.00%	100.00%	0.035966	-0.16	-0.01477	-0.12046	69280267	6467049	37798691	7.84061	0.171092
2	BANK OF AFRICA	2016	0.00%	0.00%	100.00%	100.00%	0.025061	0.002	0.000187	0.001244	55995671	4855840	31541959	7.748154	0.153949
2	BANK OF AFRICA	2017	0.00%	0.00%	100.00%	100.00%	0.023159	0.01	0.001248	0.007985	54191291	4370017	27388460	7.733929	0.159557
2	BANK OF AFRICA	2018	0.00%	0.00%	100.00%	100.00%	0.0241	0.03	0.003526	0.025693	49080859	4441624	21188115	7.690912	0.209628
2	BANK OF AFRICA	2019	0.00%	0.00%	100.00%	100.00%	0.022664		-0.04636	-0.47707	43996118	8998345	15982158	7.643414	0.563024
2	BANK OF AFRICA	2020	0.00%	0.00%	100.00%	100.00%	0.016095		-0.00478	-0.03904	45547821	8864821	17232213	7.658468	0.514433
3	BANK OF INDIA	2009	0.00%	75.30%	11.00%	100.00%	0.03752	6.800016	0.025259	0.193777	19745376	55865	649638	7.295465	0.085994
3	BANK OF INDIA	2010	0.00%	75.30%	11.00%	100.00%	0.046902	8.976972	0.032504	0.20585	20256439	61976	674539	7.306563	0.091879

3	BANK OF INDIA	2011	0.00%	75.30%	11.00%	100.00%	0.047279	10.44177	0.032796	0.226722	23352157	64438	7229142	7.368327	0.008914
3	BANK OF INDIA	2012	0.00%	75.30%	11.00%	100.00%	0.03139	7.750116	0.02285	0.139892	24876824	40913	10014941	7.395795	0.004085
3	BANK OF INDIA	2013	0.00%	75.30%	11.00%	100.00%	0.046345	13.7534	0.032858	0.198443	30721440	30568	10672752	7.487442	0.002864
3	BANK OF INDIA	2014	0.00%	81.41%	11.00%	100.00%	0.043283	13.91464	0.029714	0.16812	34370422	8399	12375611	7.536185	0.000679
3	BANK OF INDIA	2015	0.00%	81.41%	11.00%	100.00%	0.0429	12.67866	0.026278	0.15425	42162947	248694	17857613	7.624931	0.013926
3	BANK OF INDIA	2016	0.00%	81.41%	11.00%	100.00%	0.051828	18.77766	0.034318	0.172069	47815075	164313	19246080	7.679565	0.008537
3	BANK OF INDIA	2017	0.00%	81.41%	11.00%	100.00%	0.05319	23.90167	0.036882	0.17967	56630656	305235	20641381	7.753052	0.014788
3	BANK OF INDIA	2018	0.00%	81.41%	11.00%	100.00%	0.050395	22.14443	0.030868	0.146695	62689134	620255	18426559	7.797192	0.033661
3	BANK OF INDIA	2019	0.00%	81.41%	11.00%	100.00%	0.051756	26.79023	0.037432	0.150722	62543244	1212460	12870025	7.79618	0.094208
3	BANK OF INDIA	2020	0.00%	81.41%	11.00%	100.00%	0.046492	26.55715	0.03089	0.129989	75129210	996402	14229162	7.875809	0.070025
4	BARCLAYS BANK	2009	0.00%	0.34%	69.60%	68.50%	0.089582	4.5	0.036943	0.25159	164876000	5872000	93543000	8.217157	0.062773
4	BARCLAYS BANK	2010	0.00%	0.34%	69.60%	68.50%	0.090909	1.95	0.061474	0.33685	172415000	6539000	87147000	8.236575	0.075034
4	BARCLAYS BANK	2011	0.00%	0.34%	69.60%	68.50%	0.097803	1.49	0.048572	0.277624	167029000	5482000	99072000	8.222792	0.055333
4	BARCLAYS BANK	2012	0.00%	0.34%	69.60%	68.50%	0.098174	1.61	0.047292	0.295459	184825892	3771579	104204295	8.266763	0.036194
4	BARCLAYS BANK	2013	0.00%	0.34%	69.60%	68.50%	0.091222	1.4	0.036871	0.235478	206736932	3579909	118361911	8.315418	0.030245
4	BARCLAYS BANK	2014	0.00%	0.34%	73.00%	68.50%	0.086699	1.54	0.037092	0.219239	226116000	1642000	125423000	8.354331	0.013092
4	BARCLAYS BANK	2015	0.00%	0.34%	73.00%	68.50%	0.08464	1.55	0.039456	0.239576	241152000	2050000	145379000	8.382291	0.014101
4	BARCLAYS BANK	2016	0.00%	0.34%	73.00%	68.50%	0.086057	1.31	0.105053	0.647678	259525000	11472300	168510000	8.414179	0.068081
4	BARCLAYS BANK	2017	0.00%	0.34%	73.00%	68.50%	0.080034	1.23	0.024551	0.153345	272073000	12614723	168397417	8.434685	0.07491
4	BARCLAYS BANK	2018	0.00%	0.34%	73.00%	68.50%	0.067494	1.32	0.021957	0.164636	325362737	13910314	177353969	8.512368	0.078432
4	BARCLAYS BANK	2019	0.00%	0.34%	73.00%	68.50%	0.061818	1.32	0.019143	0.162467	374109200	13518692	194894941	8.572998	0.069364
4	BARCLAYS BANK	2020	0.00%	0.34%	73.00%	68.50%	0.0616	0.69	0.009905	0.083244	377935772	17099144	208854694	8.577418	0.081871
5	CBA	2009	0.00%	13.60%	49.00%	9.80%	0.05609	6.25	0.021477	0.20499	65687419	1794562	34478744	7.817482	0.052048
5	CBA	2010	0.00%	13.60%	49.00%	9.80%	0.005678	9.18	0.027477	0.250562	75459117	1892475	38642625	7.877712	0.048974
5	CBA	2011	0.00%	13.60%	49.00%	9.80%	0.035015	7.38	0.014132	0.156482	118300651	1950993	39609515	8.072987	0.049256
5	CBA	2012	0.00%	13.60%	49.00%	9.80%	0.055965	12.71	0.032956	0.242595	94771471	2015858	42504096	7.976678	0.047427
5	CBA	2013	0.00%	13.60%	49.00%	9.80%	0.044134	14.15	0.027841	0.252873	124881964	2379864	57180199	8.0965	0.04162
5	CBA	2014	0.00%	13.60%	49.00%	9.80%	0.035121	15.16	0.02147	0.211381	175808828	3770428	89362297	8.245041	0.042193
5	CBA	2015	0.00%	14.60%	52.00%	14.30%	0.057502	13.52	0.018099	0.158196	198484270	3364845	103519861	8.297726	0.032504

5	CBA	2016	0.00%	14.60%	52.00%	14.30%	0.073072	23.34	0.031847	0.244476	210877927	6160298	100314461	8.324031	0.06141
5	CBA	2017	0.00%	14.60%	52.00%	14.30%	0.034586	19.76	0.024775	0.180121	229525229	7798050	101409798	8.36083	0.076896
5	CBA	2018	0.00%	14.60%	52.00%	14.30%	0.035633	21.07	0.026094	0.179487	232317119	9271261	110736012	8.366081	0.083724
5	CBA	2019	0.00%	14.60%	52.00%	14.30%	0.005974		0.066095	0.098333	907644	15166	208105	5.957916	0.072877
5	CBA	2020					0.02462		0.171675	0.219117	862871	8233	94486	5.935946	0.087135
6	CFC STANBIC	2009	0.00%	0.00%	46.70%	84.00%	0.036436	0.22	0.000281	0.001766	127690950	1745210	70922412	8.10616	0.024607
6	CFC STANBIC	2010	0.00%	0.00%	46.70%	84.00%	0.034338	5.86	0.001837	0.016978	140020202	1887696	75224630	8.146191	0.025094
6	CFC STANBIC	2011	0.00%	0.00%	46.70%	84.00%	0.041657	5.99	0.000828	0.008469	145041100	861522	94884596	8.161491	0.00908
6	CFC STANBIC	2012	0.00%	0.00%	46.70%	84.00%	0.045686	9.9	0.021017	0.110492	143212155	1387522	78483828	8.15598	0.017679
6	CFC STANBIC	2013	0.00%	0.00%	56.00%	84.00%	0.043985	20.07	0.029046	0.221844	170726460	1784847	103847691	8.232301	0.017187
6	CFC STANBIC	2014	0.00%	0.00%	56.00%	84.00%	0.048918	32.12	0.031974	0.205624	171347152	3023730	101210110	8.233877	0.029876
6	CFC STANBIC	2015	0.00%	0.00%	56.00%	84.00%	0.044629	12.41	0.023534	0.127871	208451915	4858394	128163157	8.319006	0.037908
6	CFC STANBIC	2016	0.00%	0.00%	56.00%	84.00%	0.050586	11.18	0.020582	0.110077	214682729	5863757	132576604	8.331797	0.044229
6	CFC STANBIC	2017	0.00%	0.00%	60.00%	84.00%	0.042793	27.62	0.017325	0.100324	248738719	8727924	143279444	8.395743	0.060915
6	CFC STANBIC	2018	0.00%	0.00%	60.00%	84.00%	0.041723	36.21	0.021983	0.178547	280953012	16644228	146604117	8.448634	0.113532
6	CFC STANBIC	2019	0.00%	0.00%	60.00%	84.00%	0.043526	36.48	0.021257	0.159789	292705136	19345012	152813955	8.46643	0.126592
6	CFC STANBIC	2020	0.00%	0.00%	60.00%	84.00%	0.038146	30.63	0.016377	0.124805	318986177	25038324	158182120	8.503772	0.158288
7	CITIBANK	2009	0.00%	0.00%	100.00%	100.00%	0.043679	0.8564	0.027859	0.127102	68374519	303954	22594874	7.834894	0.013452
7	CITIBANK	2010	0.00%	0.00%	100.00%	100.00%	0.042842	0.7327	0.033329	0.153308	71935739	309528	27693464	7.856945	0.011177
7	CITIBANK	2011	0.00%	0.00%	100.00%	100.00%	0.041765	0.641989	0.039415	0.194689	74646417	405277	28451457	7.873009	0.014245
7	CITIBANK	2012	0.00%	0.00%	100.00%	100.00%	0.077218	0.966313	0.063648	0.255302	69579795	428402	23331003	7.842483	0.018362
7	CITIBANK	2013	0.00%	0.00%	100.00%	100.00%	0.058895	0.654288	0.04209	0.187836	71242659	436969	24337983	7.85274	0.017954
7	CITIBANK	2014	0.00%	0.00%	100.00%	100.00%	0.057239	0.533074	0.03077	0.133073	79397809	881135	24199843	7.899809	0.036411
7	CITIBANK	2015	0.00%	0.00%	100.00%	100.00%	0.06442	0.742086	0.038583	0.175242	88147289	1511839	29498414	7.945209	0.051252
7	CITIBANK	2016	0.00%	0.00%	100.00%	100.00%	0.060003	0.7489	0.033218	0.174856	103323540	804804	27436980	8.014199	0.029333
7	CITIBANK	2017	0.00%	2.50%	100.00%	100.00%	0.055161	0.853248	0.039808	0.193803	98231911	1723695	37187236	7.992253	0.046352
7	CITIBANK	2018	0.00%	2.50%	100.00%	100.00%	0.061903	0.689895	0.03692	0.162897	85638665	819447	26883651	7.93267	0.030481
7	CITIBANK	2019	0.00%	2.50%	100.00%	100.00%	0.014722	1.82	0.005171	0.075212	356953394		247777734	8.552612	0
7	CITIBANK	2020	0.00%	2.50%	100.00%	100.00%	0.010168	1.05	0.002661	0.042622	401448589		293219676	8.60363	0

8	CONSOLIDATED BANK	2009	86.00%	0.35%	66.20%	0.00%	0.074579	4.06	0.011732	0.083716	6898919	591180	3868472	6.838781	0.15282
8	CONSOLIDATED BANK	2010	86.00%	0.35%	66.20%	0.00%	0.060814	8.66	0.016902	0.134197	10204682	732918	6047276	7.008799	0.121198
8	CONSOLIDATED BANK	2011	86.00%	0.35%	66.20%	4.30%	0.056461	7.52	0.009781	0.104421	15318148	813248	9197024	7.185206	0.088425
8	CONSOLIDATED BANK	2012	86.00%	0.35%	66.20%	4.30%	0.039486	-1.84	0.002034	0.002034	18064213	1319631	10077068	7.256819	0.130954
8	CONSOLIDATED BANK	2013	86.00%	0.35%	66.20%	4.30%	0.064073	-5.48	0.006503	0.006503	16778631	1660831	10855492	7.224757	0.152995
8	CONSOLIDATED BANK	2014	86.00%	0.35%	77.80%	4.30%	0.060565	-14.14	-0.01868	-0.17963	15077051	2810500	9212581	7.178316	0.305072
8	CONSOLIDATED BANK	2015	86.00%	0.35%	77.80%	4.30%	0.072076	0.87	0.003143	0.027502	14135528	1957862	9221256	7.150312	0.212321
8	CONSOLIDATED BANK	2016	86.00%	0.35%	77.80%	6.40%	0.063665	-4.70525	-0.01519	-0.15064	13917895	2037505	9161484	7.143574	0.222399
8	CONSOLIDATED BANK	2017	86.00%	0.35%	77.80%	6.40%	0.037043	-7.38	-0.02495	-0.31418	13455744	1720171	8421072	7.128908	0.20427
8	CONSOLIDATED BANK	2018	86.00%	0.35%	84.00%	6.40%	0.049895	-7.72	-0.0419	-0.5836	12887332	1830300	8429659	7.110163	0.217126
8	CONSOLIDATED BANK	2019	86.00%	0.35%	84.00%	6.40%	0.045632		0.044684	0.265129	11865609	2632023	7369033	7.07429	0.357173
8	CONSOLIDATED BANK	2020	86.00%	0.35%	84.00%	6.40%	0.049935		0.021502	0.150821	12885742	2436487	8465014	7.110109	0.28783
9	COOP BANK	2009	0.00%	1.89%	86.00%	0.00%	0.063732	0.85	0.026816	0.182178	110678091	4172700	62274194	8.044062	0.067005
9	COOP BANK	2010	0.00%	1.89%	86.00%	0.00%	0.061569	1.31	0.029679	0.222406	154339991	3040113	86618311	8.188478	0.035098
9	COOP BANK	2011	0.00%	1.89%	86.00%	0.00%	0.070739	1.48505	0.030913	0.247303	167772389	5207834	109408815	8.22472	0.0476
9	COOP BANK	2012	0.00%	1.89%	93.50%	0.00%	0.079715	1.748915	0.036709	0.253027	199662974	6203278	119087748	8.300298	0.05209
9	COOP BANK	2013	0.00%	1.89%	93.50%	0.00%	0.081246	2.14	0.038795	0.249051	228874484	6103463	137051537	8.359597	0.044534
9	COOP BANK	2014	0.00%	2.10%	93.50%	0.00%	0.074997	1.71	0.029542	0.197193	282689098	7982045	178978586	8.451309	0.044598
9	COOP BANK	2015	0.00%	2.10%	93.50%	0.00%	0.06775	2.31	0.034177	0.23742	342499809	8189196	308571920	8.53466	0.026539

9	COOP BANK	2016	0.00%	2.10%	97.72%	0.00%	0.083837	2.64	0.03603	0.209109	351828577	11274174	232307329	8.546331	0.048531
9	COOP BANK	2017	0.00%	2.28%	97.72%	0.00%	0.072776	1.98	0.030393	0.170541	382829640	18713757	252361773	8.583006	0.074154
9	COOP BANK	2018	0.00%	2.28%	97.72%	0.00%	0.074415	2.11	0.030392	0.181634	408303625	28952802	243546383	8.610983	0.11888
9	COOP BANK	2019	0.00%	2.28%	97.72%	0.00%	0.068861		0.031706	0.184936	449616472	31155503	265332776	8.652842	0.11742
9	COOP BANK	2020	0.00%	2.28%	97.72%	0.00%	0.069238		0.027614	0.160277	496822948	51781184	280522176	8.696202	0.184589
10	CREDIT BANK	2009	0.00%	9.70%	23.50%	0.00%	0.070233	11	0.015772	0.079429	3664947	289787	1880943	6.564068	0.154065
10	CREDIT BANK	2010	0.00%	9.70%	23.50%	0.00%	0.063854	4	0.007459	0.035661	4530094	500894	1926918	6.656107	0.259946
10	CREDIT BANK	2011	0.00%	9.70%	23.50%	0.00%	0.061539	6	0.008727	0.049136	5394064	440337	2883261	6.731916	0.152722
10	CREDIT BANK	2012	0.00%	11.30%	23.50%	0.00%	0.062709	7	0.010873	0.059102	6407485	387277	3112099	6.806688	0.124442
10	CREDIT BANK	2013	0.00%	11.30%	27.60%	0.00%	0.071062	3.5	0.007224	0.042658	7308855	344829	4328080	6.863849	0.079673
10	CREDIT BANK	2014	0.00%	11.30%	27.60%	2.50%	0.061963	-6.1	-0.01035	-0.07964	8864537	586252	5527640	6.947656	0.106058
10	CREDIT BANK	2015	0.00%	11.30%	27.60%	2.50%	0.060139	-4.5	-0.00581	-0.04296	10287085	514747	7087728	7.012292	0.072625
10	CREDIT BANK	2016	0.00%	11.30%	27.60%	2.50%	0.065784	5.4	0.008674	0.043031	12201968	676221	7899394	7.08643	0.085604
10	CREDIT BANK	2017	0.00%	14.96%	34.00%	2.50%	0.05483	5.43	0.008977	0.048721	14465074	876544	9698546	7.160321	0.090379
10	CREDIT BANK	2018	0.00%	14.96%	34.00%	3.90%	0.052433	10.02	0.013537	0.084189	17805424	1112560	13031250	7.250552	0.085376
10	CREDIT BANK	2019	0.00%	14.96%	34.00%	3.90%	0.042758		0.00976	0.070067	21540739	1592274	15226683	7.333261	0.104571
10	CREDIT BANK	2020	0.00%	14.96%	34.00%	3.90%	0.047381		-0.00273	-0.0196	23145338	2016878	15631035	7.364464	0.12903
11	DEVELOPMENT BANK	2009	90.80%	0.00%	0.00%	7.90%	0.067398	9.87	0.014171	0.084561	11843593	649023	502587	7.073483	1.291364
11	DEVELOPMENT BANK	2010	90.80%	0.00%	0.00%	7.90%	0.05753	9.21	0.015027	0.107138	10644841	785980	5732795	7.027139	0.137102
11	DEVELOPMENT BANK	2011	90.80%	0.00%	0.00%	12.30%	0.06381	6.28	0.009478	0.06966	11517988	865783	6247942	7.061377	0.138571
11	DEVELOPMENT BANK	2012	89.30%	0.00%	0.00%	12.30%	0.048838	4.25	0.005501	0.044962	13411458	980548	7300320	7.127476	0.134316
11	DEVELOPMENT BANK	2013	89.30%	0.00%	0.00%	12.30%	0.040972	10.9	0.012163	0.103495	15574646	1189931	8646163	7.192418	0.137625
11	DEVELOPMENT BANK	2014	89.30%	0.00%	0.00%	12.30%	0.040357	12.7	0.013019	0.079569	16944142	1246349	9225833	7.22902	0.135093
11	DEVELOPMENT	2015	89.30%	0.00%	0.00%	12.30%	0.032132	2.33	0.007178	0.042769	16942714	1869831	8043938	7.228983	0.232452

	BANK														
11	DEVELOPMENT BANK	2016	89.30%	0.00%	0.00%	10.70%	0.029445	1.88	0.003638	0.020571	16418382	2594459	8733212	7.21533	0.29708
11	DEVELOPMENT BANK	2017	89.30%	0.00%	0.00%	10.70%	0.024266	0.075977	0.001618	0.009012	16319925	2310400	9199779	7.212718	0.251136
11	DEVELOPMENT BANK	2018	89.30%	0.00%	0.00%	10.70%	0.027508	0.329338	0.007469	0.039858	15323111	2879070	8386697	7.185347	0.34329
11	DEVELOPMENT BANK	2019	89.30%	0.00%	0.00%	10.70%	0.026337		0.070264	0.273163	15358069	3365446	7911046	7.186337	0.425411
11	DEVELOPMENT BANK	2020	89.30%	0.00%	0.00%	10.70%	0.021037		0.001195	0.005383	17222212	3419763	8609289	7.236089	0.397218
12	DTB	2009	0.00%	0.00%	32.40%	15.60%	0.07465	7.67	0.028728	0.216249	47146767	6129053	29700279	7.673452	0.206363
12	DTB	2010	0.00%	0.00%	32.40%	15.60%	0.058615	10.52	0.035118	0.255436	58605823	682849	37850277	7.767941	0.018041
12	DTB	2011	0.00%	0.00%	32.40%	15.60%	0.060894	11.48	0.02901	0.216746	77453024	661705	50943685	7.889038	0.012989
12	DTB	2012	0.00%	0.00%	32.40%	16.15%	0.072652	14.75	0.032469	0.20625	94511818	809946	59930459	7.975486	0.013515
12	DTB	2013	0.00%	0.00%	32.40%	16.15%	0.070028	17.89	0.035553	0.218539	114136429	972023	75292211	8.057424	0.01291
12	DTB	2014	0.00%	0.00%	33.00%	16.15%	0.062635	17.9	0.029413	0.161044	141175794	1119140	94059269	8.14976	0.011898
12	DTB	2015	0.00%	0.00%	33.00%	16.15%	0.058848	18	0.0251	0.159782	190947903	3269570	125817859	8.280915	0.025987
12	DTB	2016	0.00%	0.00%	33.00%	16.15%	0.060109	23.1	0.0252	0.168861	244123818	4486767	136685924	8.38761	0.032825
12	DTB	2017	0.00%	0.00%	33.00%	16.15%	0.053658	20.23	0.020363	0.127888	270081538	10597748	148515793	8.431495	0.071358
12	DTB	2018	0.00%	0.00%	33.00%	16.15%	0.05318	20.7	0.020556	0.121286	281515703	9710643	146781681	8.449503	0.066157
12	DTB	2019	0.00%	0.00%	33.00%	16.15%	0.044381		0.019895	0.109901	287250595	12891650	149501314	8.458261	0.086231
12	DTB	2020	0.00%	0.00%	33.00%	16.15%	0.038453		0.009757	0.056373	312189185	19747394	154998068	8.494418	0.127404
13	ECO BANK	2009	0.00%	0.00%	0.00%	75.00%	0.025807	-15.8	-0.05708	-0.37068	13949401	1939372	6444336	7.144556	0.300942
13	ECO BANK	2010	0.00%	0.00%	0.00%	75.00%	0.03413	2.56	0.004653	0.025003	26892184	3409032	9693276	7.429626	0.35169
13	ECO BANK	2011	0.00%	0.00%	0.00%	75.00%	0.025958	2.75	0.007428	0.117079	27210496	2435103	11380592	7.434736	0.21397
13	ECO BANK	2012	0.00%	0.00%	0.00%	75.00%	0.00139	-9.2	-0.03323	-0.52809	31771339	707596	13968266	7.502036	0.050657
13	ECO BANK	2013	0.00%	0.00%	0.00%	96.67%	0.024418	5.6	0.023895	0.260114	36907136	2193226	18459837	7.56711	0.118811
13	ECO BANK	2014	0.00%	0.00%	0.00%	96.67%	0.021717	1.3	0.006971	0.040906	45934458	2460719	22982094	7.662139	0.107071
13	ECO BANK	2015	0.00%	0.00%	0.00%	96.67%	0.032612	0.37	0.001724	0.011953	52426513	2444338	29621116	7.719551	0.08252

13	ECO BANK	2016	0.00%	0.00%	0.00%	96.67%	0.006145	-8.3	-0.04295	-0.27697	47123839	5358806	24473512	7.673241	0.218964
13	ECO BANK	2017	0.00%	0.00%	0.00%	100.00%	0.041513	-4.6	-0.02086	-0.17323	53455760	8286628	16370967	7.727995	0.506178
13	ECO BANK	2018	0.00%	0.00%	0.00%	100.00%	0.031553	0.3	0.001294	0.010999	54463878	3191981	13022796	7.736109	0.245107
13	ECO BANK	2019	0.00%	0.00%	0.00%	100.00%	0.02798		0.001689	0.019387	75377850	4781738	21377402	7.877244	0.223682
13	ECO BANK	2020	0.00%	0.00%	0.00%	100.00%	0.024956		-0.00022	-0.00293	94428325	4377195	24278479	7.975102	0.180291
14	EQUITY BANK	2009	0.00%	23.50%	85.00%	0.00%	0.085767	1.23	0.047281	0.195528	96511725	4565118	59868232	7.98458	0.076253
14	EQUITY BANK	2010	0.00%	23.50%	85.00%	0.00%	0.082579	2.04	0.056422	0.266868	133889997	3470580	72902021	8.126748	0.047606
14	EQUITY BANK	2011	0.00%	23.50%	85.00%	0.00%	0.082647	2.79	0.0526	0.301152	196293896	3250727	113823792	8.292907	0.028559
14	EQUITY BANK	2012	0.00%	23.50%	85.00%	0.00%	0.10245	2.97	0.050952	0.257708	215829300	4018405	122410013	8.33411	0.032827
14	EQUITY BANK	2013	0.00%	23.50%	85.00%	0.00%	0.099456	3.41	0.053074	0.24941	238194354	8188895	152028916	8.376931	0.053864
14	EQUITY BANK	2014	0.00%	0.00%	100.00%	0.00%	0.094741	4.55	0.060974	0.423724	276115727	7469299	187976229	8.441091	0.039735
14	EQUITY BANK	2015	0.00%	0.00%	100.00%	0.00%	0.086388	539.18	0.047389	0.340967	341329318	6832277	225036662	8.533174	0.030361
14	EQUITY BANK	2016	0.00%	0.00%	100.00%	0.00%	0.092323	507.27	0.040074	0.290751	379748996	15457320	213805548	8.579497	0.072296
14	EQUITY BANK	2017	0.00%	0.00%	100.00%	0.00%	0.072002	544.6	0.040202	0.263916	406402487	14757622	214484733	8.608956	0.068805
14	EQUITY BANK	2018	0.00%	0.00%	100.00%	0.00%	0.072988	560.1	0.038318	0.277337	438508784	17063515	223565307	8.641978	0.076325
14	EQUITY BANK	2019	0.00%	0.00%	100.00%	0.00%	0.06781		0.036932	0.268096	507525237	26184843	276863043	8.705458	0.094577
14	EQUITY BANK	2020	0.00%	0.00%	100.00%	0.00%	0.059943		0.020975	0.161528	667649767	42824644	313065151	8.824549	0.136791
15	FAMILY BANK	2009	0.00%	4.30%	52.50%	0.00%	0.105101	1.2	0.016419	0.119217	13453266.1	493516	7675806.2	7.128828	0.064295
15	FAMILY BANK	2010	0.00%	4.30%	52.50%	25.00%	0.1006	1.62	0.019368	0.125028	20188377.8	1000179	10208136.5	7.305101	0.097979
15	FAMILY BANK	2011	0.00%	4.30%	52.50%	25.00%	0.090547	1.46	0.013638	0.106689	26001754	1824592	16332359	7.415003	0.111716
15	FAMILY BANK	2012	0.00%	4.30%	52.50%	25.00%	0.106191	1.94	0.017451	0.11126	30985096	2772892	17868745	7.491153	0.155181
15	FAMILY BANK	2013	0.00%	4.30%	52.50%	0.00%	0.102306	1.09	0.028193	0.205486	43500989	2316669	27943360	7.638499	0.082906
15	FAMILY BANK	2014	0.00%	4.30%	52.50%	0.00%	0.086887	1.6	0.028806	0.167655	61812663	2847219	37925476	7.791077	0.075074
15	FAMILY BANK	2015	0.00%	6.00%	57.00%	0.00%	0.078734	1.55	0.023853	0.162379	81190214	3514571	55853882	7.909504	0.062924
15	FAMILY BANK	2016	0.00%	6.00%	57.00%	0.00%	0.096483	0.27	0.004771	0.026254	69432374	7014631	50163555	7.841562	0.139835
15	FAMILY BANK	2017	0.00%	6.00%	57.00%	0.00%	0.063341	-0.81	-0.01462	-0.08697	69050943	9477932	43471853	7.83917	0.218025
15	FAMILY BANK	2018	0.00%	6.00%	57.00%	0.00%	0.064781	0.19	0.00351	0.020553	66909838	8137866	44113093	7.82549	0.184477
15	FAMILY BANK	2019	0.00%	6.00%	57.00%	0.00%	0.063315		0.011362	0.072207	78857125	8244017	50594439	7.896841	0.162943
15	FAMILY BANK	2020	0.00%	6.00%	57.00%	0.00%	0.070714		0.011878	0.081757	90590626	9390878	56579798	7.957083	0.165976

16	FIRST COMMUNITY BANK	2009	0.00%	27.60%	0.00%	34.00%	0.115	0.15841	0.0342	0.035556	138956	0	119765	5.142877	0
16	FIRST COMMUNITY BANK	2010	0.00%	27.60%	0.00%	34.00%	0.104023	0.17304	0.036305	0.036053	142987	0	120897	5.155297	0
16	FIRST COMMUNITY BANK	2011	0.00%	27.60%	14.80%	33.89%	0.109433	0.11	0.030719	0.030719	148767	0	121332	5.172507	0
16	FIRST COMMUNITY BANK	2012	0.00%	27.60%	14.80%	33.89%	4.715168	-0.01	1.41571	1.41571	170448	749199	165648	5.231592	4.522838
16	FIRST COMMUNITY BANK	2013	0.00%	27.60%	14.80%	33.89%	0.075221	0.44	0.011694	0.109249	11305399	542260	7211504	7.053286	0.075194
16	FIRST COMMUNITY BANK	2014	0.00%	32.86%	14.80%	33.89%	0.059349	0.14	0.003301	0.033232	15278026	1517821	9765509	7.184067	0.155427
16	FIRST COMMUNITY BANK	2015	0.00%	32.86%	14.80%	33.89%	0.071302	-0.03	-0.00072	-0.00656	14612851	2776851	10940003	7.164735	0.253825
16	FIRST COMMUNITY BANK	2016	0.00%	32.86%	16.56%	33.89%	0.067305	-0.14	-0.00373	-0.03579	14962089	3852974	10939122	7.174992	0.35222
16	FIRST COMMUNITY BANK	2017	0.00%	32.86%	16.56%	33.89%	0.046213	0.39	0.008744	0.088811	17359968	4398584	9726807	7.239549	0.452213
16	FIRST COMMUNITY BANK	2018	0.00%	32.86%	16.56%	33.89%	0.040257	-0.54	-0.01186	-0.16683	17880462	4940176	9027389	7.252379	0.547243
16	FIRST COMMUNITY BANK	2019	0.00%	32.86%	16.56%	33.89%	0.040051		0.010121	0.13059	18864842	4699124	9909696	7.275653	0.474195

16	FIRST COMMUNITY BANK	2020	0.00%	32.86%	16.56%	33.89%	0.040302		0.008459	0.090528	21946547	5257704	12618737	7.341366	0.416658
17	GUARANTY TRUST BANK	2009	0.00%	0.00%	0.00%	0.00%	0.078882	117.93	0.008912	0.008912	12278678	865753	5937140	7.089152	0.14582
17	GUARANTY TRUST BANK	2010	0.00%	0.00%	0.00%	0.00%	0.081614	253.46	0.0095	0.0095	14112365	1072386	6718235	7.1496	0.159623
17	GUARANTY TRUST BANK	2011	0.00%	0.00%	0.00%	0.00%	0.09134	516.94	0.021259	0.202557	14630459	450666	2776704	7.165258	0.162302
17	GUARANTY TRUST BANK	2012	0.00%	0.00%	13.20%	0.00%	0.081849	727.22	0.027209	0.186408	17152445	329810	8742625	7.234326	0.037724
17	GUARANTY TRUST BANK	2013	0.00%	0.00%	13.20%	70.00%	0.043156	273.3	0.012922	0.054393	25638050	458639	10303477	7.408885	0.044513
17	GUARANTY TRUST BANK	2014	0.00%	0.00%	13.20%	70.00%	0.043894	418.1	0.016175	0.074479	32991926	471969	12440594	7.518408	0.037938
17	GUARANTY TRUST BANK	2015	0.00%	2.40%	14.60%	70.00%	0.057883	368.4	0.016008	0.059477	29374062	570248	12422326	7.467964	0.045905
17	GUARANTY TRUST BANK	2016	0.00%	2.40%	14.60%	70.00%	0.059173	350.1	0.015086	0.053408	29619072	993678	12906196	7.471571	0.076992
17	GUARANTY TRUST BANK	2017	0.00%	2.40%	14.60%	70.00%	0.048434	150.7	0.006986	0.022419	27627849	1420820	13203981	7.441347	0.107605
17	GUARANTY TRUST BANK	2018	0.00%	2.40%	14.60%	70.00%	0.050859	192	0.009712	0.029094	25323367	2525574	12669666	7.403521	0.19934
17	GUARANTY TRUST BANK	2019	0.00%	2.40%	14.60%	70.00%	0.047852		0.012846	0.13631	29082395	2746725	13544305	7.46363	0.202796
17	GUARANTY TRUST BANK	2020	0.00%	2.40%	14.60%	70.00%	0.047422		0.005874	0.078688	31267343	3268625	13081720	7.495091	0.249862
18	GUARDIAN BANK	2009	0.00%	0.00%	100.00%	0.00%	0.052774	8.628787	0.017122	0.172905	11348656	672869	7904567	7.054944	0.085124
18	GUARDIAN BANK	2010	0.00%	0.00%	100.00%	0.00%	0.051384	11.99681	0.022915	0.225769	11789458	701456	8508756	7.071494	0.082439
18	GUARDIAN BANK	2011	0.00%	0.00%	100.00%	0.00%	0.053586	9.520311	0.016908	0.167559	12679734	698345	8118564	7.10311	0.086018

18	GUARDIAN BANK	2012	0.00%	0.00%	100.00%	0.00%	0.056259	10.68709	0.01855	0.179079	12973476	727894	8176456	7.113056	0.089023
18	GUARDIAN BANK	2013	0.00%	0.00%	100.00%	0.00%	0.055949	12.23	0.020979	0.192323	13124071	730988	8335794	7.118069	0.087693
18	GUARDIAN BANK	2014	0.00%	0.00%	100.00%	0.00%	0.057558	11.6	0.017557	0.154232	14880070	587344	9441563	7.172605	0.062208
18	GUARDIAN BANK	2015	0.00%	0.00%	100.00%	0.00%	0.063591	10.18	0.015401	0.120763	14890350	819672	9223218	7.172905	0.088871
18	GUARDIAN BANK	2016	0.00%	0.00%	100.00%	0.00%	0.065985	10.22	0.015649	0.103914	14705350	786925	8974527	7.167475	0.087684
18	GUARDIAN BANK	2017	0.00%	0.00%	100.00%	0.00%	0.051711	7.1	0.010126	0.067389	15802759	1121770	9616965	7.198733	0.116645
18	GUARDIAN BANK	2018	0.00%	0.00%	100.00%	0.00%	0.056518	10.0169	0.013936	0.088211	16185964	960439	9028027	7.209139	0.106384
18	GUARDIAN BANK	2019	0.00%	0.00%	100.00%	0.00%	0.047303		0.011208	0.067009	16386450	691838	9102560	7.214485	0.076005
18	GUARDIAN BANK	2020	0.00%	0.00%	100.00%	0.00%	0.043577		0.005553	0.039934	16784774	873016	7894419	7.224915	0.110586
19	GULF AFRICAN BANK	2009	0.00%	0.00%	90.10%	95.00%	0.060754	1.131536	0.001588	0.117033	8904378	1076935	6078453	6.949604	0.177173
19	GULF AFRICAN BANK	2010	0.00%	0.00%	90.80%	95.00%	0.061337	5.9	0.007702	0.060379	9594061	1169134	6270884	6.982002	0.186438
19	GULF AFRICAN BANK	2011	0.00%	0.00%	90.80%	95.00%	0.061654	7.6	0.007382	0.072269	12915174	1247132	7439551	7.1111	0.167635
19	GULF AFRICAN BANK	2012	0.00%	0.00%	90.80%	95.00%	0.086883	19.4	0.017861	0.155131	13561818	1634261	9446582	7.132318	0.173
19	GULF AFRICAN BANK	2013	0.00%	0.00%	90.80%	95.00%	0.080105	15.9	0.017782	0.106286	16053971	1821453	10665498	7.205582	0.17078
19	GULF AFRICAN BANK	2014	0.00%	0.00%	90.80%	95.00%	0.078565	29.9	0.020319	0.127554	19753647	1504583	13790646	7.295647	0.109102
19	GULF AFRICAN BANK	2015	0.00%	0.00%	90.80%	95.00%	0.081342	39.9	0.029567	0.188452	24713782	1397624	15427705	7.392939	0.090592

19	GULF AFRICAN BANK	2016	0.00%	0.00%	90.80%	95.00%	0.075496	27.21	0.01835	0.113884	27156264	1617177	16193046	7.43387	0.099869
19	GULF AFRICAN BANK	2017	0.00%	0.00%	90.80%	95.00%	0.062364	8.39	0.004906	0.034767	31316228	1961704	19384156	7.495769	0.101201
19	GULF AFRICAN BANK	2018	0.00%	0.00%	90.80%	95.00%	0.066175	7.2	0.003949	0.029452	33325575	2571643	22605853	7.522778	0.11376
19	GULF AFRICAN BANK	2019	0.00%	0.00%	90.80%	95.00%	0.05253		0.004755	0.03603	35122982	3613126	22673040	7.545591	0.159358
19	GULF AFRICAN BANK	2020	0.00%	0.00%	90.80%	95.00%	0.057516		0.010455	0.078286	37652622	4028256	20698572	7.575795	0.194615
20	HABIB BANK (HBL)	2009	0.00%	0.00%	100.00%	100.00%	0.050739	12.28	0.014978	0.155491	821127519	42309377	432283588	8.914411	0.097874
20	HABIB BANK (HBL)	2010	0.00%	0.00%	100.00%	100.00%	0.051755	15.58	0.017601	0.174399	887052411	46677077	434998560	8.947949	0.107304
20	HABIB BANK (HBL)	2011	0.00%	0.00%	100.00%	100.00%	0.049491	18.3	0.019598	0.203793	1139554205	47780456	457367656	9.056735	0.104468
20	HABIB BANK (HBL)	2012	0.00%	0.00%	100.00%	100.00%	0.035869	18.36	0.013883	0.168041	1610308572	56095106	499817906	9.206909	0.112231
20	HABIB BANK (HBL)	2013	0.00%	0.00%	100.00%	100.00%	0.032297	15.59	0.013425	0.161668	1715271378	66106644	563700737	9.234333	0.117273
20	HABIB BANK (HBL)	2014	0.00%	0.00%	100.00%	100.00%	0.037004	21.63	0.017043	0.187061	1867003389	77290030	595295176	9.271145	0.129835
20	HABIB BANK (HBL)	2015	0.00%	0.00%	100.00%	100.00%	0.035236	23.93	0.015823	0.221668	2218432983	66989249	637383859	9.346046	0.1051
20	HABIB BANK (HBL)	2016	0.00%	0.00%	100.00%	100.00%	0.032687	23.23	0.013643	0.202681	2507182331	85258822	748466297	9.399186	0.113911
20	HABIB BANK (HBL)	2017	0.00%	0.00%	100.00%	100.00%	0.030351	5.79	0.003282	0.04776	2696218064	75845393	851502420	9.430755	0.089072
20	HABIB BANK (HBL)	2018					0.026993	8.22	0.004112	0.063721	3025853150	80231013	1080440220	9.480848	0.074258
21	HABIB BANK (AG ZURICH)	2009	0.00%	75.00%	0.00%	100.00%	0.032175	0.529891	0.014108	0.140595	17034923	199045	2490784	7.23134	0.079913

21	HABIB BANK (AG ZURICH)	2010	0.00%	75.00%	0.00%	100.00%	0.03101	0.453107	0.011406	0.113677	18017328	201043	3308945	7.25569	0.060757
21	HABIB BANK (AG ZURICH)	2011	0.00%	75.00%	0.00%	100.00%	0.044122	0.563965	0.013531	0.134317	18903674	278065	4289043	7.276546	0.064831
21	HABIB BANK (AG ZURICH)	2012	0.00%	75.00%	0.00%	100.00%	0.035834	0.518737	0.011839	0.117617	19872347	250274	3807848	7.298249	0.065726
21	HABIB BANK (AG ZURICH)	2013	0.00%	75.00%	0.00%	100.00%	0.033116	0.491537	0.011203	0.106711	19900376	302067	4567061	7.298861	0.06614
21	HABIB BANK (AG ZURICH)	2014	0.00%	75.00%	0.00%	100.00%	0.037092	0.459033	0.009754	0.081157	20038597	389034	4890765	7.301867	0.079545
21	HABIB BANK (AG ZURICH)	2015	0.00%	75.00%	0.00%	100.00%	0.041983	0.624579	0.01304	0.088919	20394576	490621	5408978	7.309515	0.090705
21	HABIB BANK (AG ZURICH)	2016	0.00%	75.00%	0.00%	100.00%	0.035796	0.58236	0.011931	0.082305	20783427	550982	5908674	7.317717	0.09325
21	HABIB BANK (AG ZURICH)	2017	0.00%	75.00%	0.00%	100.00%	0.043414	0.675126	0.013454	0.098839	21367296	570834	6184674	7.32975	0.092298
21	HABIB BANK (AG ZURICH)	2018	0.00%	75.00%	0.00%	100.00%	0.046118	0.530761	0.010501	0.074371	21520666	581154	6286399	7.332856	0.092446
21	HABIB BANK (AG ZURICH)	2019	0.00%	75.00%	0.00%	100.00%	0.032676	0.316346	0.005565	0.045322	24204929	762809	6767207	7.383904	0.112721
22	BANK OF BARODA	2009	0.00%	0.00%	85.60%	88.60%	0.045883	27.5	0.040551	0.280007	31678367	483489	11987345	7.500763	0.040333
22	BANK OF BARODA	2010	0.00%	0.00%	85.60%	88.60%	0.051872	28.16	0.043097	0.293722	32331505	512940	13434459	7.509626	0.038181
22	BANK OF BARODA	2011	0.00%	0.00%	85.60%	88.60%	0.062291	27.56	0.037162	0.276322	36700797	648851	19144038	7.564675	0.033893
22	BANK OF BARODA	2012	0.00%	0.00%	85.60%	88.60%	0.046551	27.81	0.029826	0.238974	46137777	583766	21922597	7.664057	0.026629
22	BANK OF BARODA	2013	0.00%	0.00%	85.60%	86.70%	0.058522	41.22	0.039209	0.269476	52021524	598364	23578560	7.716183	0.025377
22	BANK OF BARODA	2014	0.00%	0.00%	86.70%	86.70%	0.054504	44.8	0.035789	0.224672	61944650	1064626	28388852	7.792004	0.037502

22	BANK OF BARODA	2015	0.00%	0.00%	86.70%	86.70%	0.054384	40.94	0.029718	0.17973	68177548	2363810	31018373	7.833641	0.076207
22	BANK OF BARODA	2016	0.00%	0.00%	86.70%	86.70%	0.060192	59.55	0.035543	0.207155	82907475	3392267	36400900	7.918594	0.093192
22	BANK OF BARODA	2017	0.00%	0.00%	86.70%	86.70%	0.060079	79.28	0.040808	0.219163	96132100	2665782	42207280	7.982868	0.063159
22	BANK OF BARODA	2018	0.00%	0.00%	86.70%	86.70%	0.052329	79.41	0.031944	0.192487	123014401	3903308	41570848	8.089956	0.093895
22	BANK OF BARODA	2019	0.00%	0.00%	86.70%	86.70%	0.047001	41.35	0.028559	0.178391	143311335	4126146	46941977	8.156281	0.087899
22	BANK OF BARODA	2020	0.00%	0.00%	86.70%	86.70%	0.045848	45.7	0.027193	0.169531	166312530	6341985	47635245	8.220925	0.133136
23	HOUSING FINANCE	2009	3.60%	0.00%	55.90%	0.00%	0.062773	1.02	0.01281	0.057337	18280761	1815135	14495208	7.261994	0.125223
23	HOUSING FINANCE	2010	3.60%	0.00%	55.90%	0.00%	0.047773	1.65	0.012942	0.088893	29325842	1467815	19503400	7.46725	0.075259
23	HOUSING FINANCE	2011	3.60%	0.00%	55.90%	0.00%	0.059474	2.93	0.021112	0.14115	31972113	1579576	25222836	7.504771	0.062625
23	HOUSING FINANCE	2012	3.60%	0.00%	55.90%	0.00%	0.047927	2.98	0.016887	0.133517	40685928	2331482	30293711	7.609444	0.076963
23	HOUSING FINANCE	2013	2.41%	0.00%	55.90%	0.00%	0.054604	3.5	0.017302	0.142378	46755111	3209739	35215897	7.669829	0.091145
23	HOUSING FINANCE	2014	2.41%	0.00%	66.00%	0.00%	0.049892	3.76	0.014385	0.138645	60490833	4163451	45243539	7.78169	0.092023
23	HOUSING FINANCE	2015	2.41%	0.00%	66.00%	0.00%	0.052051	118.8	0.019253	0.14574	68808654	4092356	53021022	7.837643	0.077184
23	HOUSING FINANCE	2016	2.41%	0.00%	66.00%	0.00%	0.058212	200.25	0.014706	0.102429	68084930	6193462	54469605	7.833051	0.113705
23	HOUSING FINANCE	2017	2.41%	0.00%	66.00%	0.00%	0.049227	36.25	0.002917	0.018191	62126556	8212167	49639639	7.793277	0.165436
23	HOUSING FINANCE	2018	2.41%	0.00%	66.00%	0.00%	0.042635	-85.77	-0.00751	-0.04679	57083283	13334356	44615058	7.756509	0.298876

23	HOUSING FINANCE	2019	2.41%	0.00%	66.00%	0.00%	0.042243	1.27	0.000116	0.000694	54531890	12316003	39934816	7.736651	0.308403
23	HOUSING FINANCE	2020	2.41%	0.00%	66.00%	0.00%	0.036935	-186.35	-0.0171	-0.11298	54478060	10798847	38194786	7.736222	0.282731
24	I&M	2009	0.00%	0.00%	75.60%	0.00%	0.053165	51.26	0.027464	0.162911	44009221.2	1073198.8	24591499.9	7.643544	0.043641
24	I&M	2010	0.00%	0.00%	75.60%	0.00%	0.050627	80.32	0.033859	0.163128	62536574.7	1884404	35642515	7.796134	0.05287
24	I&M	2011	0.00%	0.00%	75.60%	0.00%	0.060666	107.65	0.04024	0.223338	76903271.4	1004454.1	46778935.3	7.885945	0.021472
24	I&M	2012	0.00%	0.00%	75.60%	0.00%	0.05028	9.84	0.03455	0.212703	119233345	1444103.1	71012960.1	8.076398	0.020336
24	I&M	2013	0.00%	0.00%	76.20%	0.00%	0.006294	11.75	0.003523	0.210109	1412005445	1522653.7	91882663.9	9.149836	0.016572
24	I&M	2014	0.00%	0.00%	76.20%	0.00%	0.058959	13.56	0.032494	0.204013	176464451	2532498	112491329	8.246657	0.022513
24	I&M	2015	0.00%	3.40%	76.20%	2.65%	0.066845	204.85	0.036601	0.224962	164822609	5467906	114927247	8.217017	0.047577
24	I&M	2016	0.00%	3.40%	77.65%	2.65%	0.074465	225.85	0.03613	0.205111	182157482	8979494	3253269	8.260447	2.760145
24	I&M	2017	0.00%	3.40%	77.65%	2.65%	0.006474	16.37	0.030254	0.154508	240110741	19484639	153018152	8.380412	0.127335
24	I&M	2018	0.00%	3.40%	77.65%	2.65%	0.054048	19.23	0.029472	0.167144	288522049	22492452	166736729	8.460179	0.134898
24	I&M	2019	0.00%	3.40%	77.65%	2.65%	0.049192	12.47	0.034155	0.186506	315290674	21310499	175329426	8.498711	0.121545
24	I&M	2020	0.00%	3.40%	77.65%	2.65%	0.043562	9.77	0.023492	0.131058	358099793	23595463	187391266	8.554004	0.125915
25	JAMII BORA	2009	0.00%	0.00%	73.25%	0.00%	0.079589	0.06423	0.016787	0.047648	5376934	197453	2538765	6.730535	0.077775
25	JAMII BORA	2010	0.00%	0.00%	73.25%	0.00%	0.076862	0.074409	0.017534	0.052693	5963456	208342	2743678	6.775498	0.075935
25	JAMII BORA	2011	0.00%	0.00%	73.25%	0.00%	0.058947	0.081558	0.01699	0.05793	6745854	237467	2845378	6.829037	0.083457
25	JAMII BORA	2012	0.00%	0.00%	73.25%	0.00%	0.068666	0.084985	0.017315	0.054419	6897354	257832	3706476	6.838683	0.069563
25	JAMII BORA	2013	0.00%	0.00%	73.25%	0.00%	0.055383	0.066811	0.013393	0.041716	7010323	281635	3809603	6.845738	0.073928
25	JAMII BORA	2014	0.00%	0.00%	73.25%	0.00%	0.043925	0.014009	0.001501	0.006339	13117892	531139	6189800	7.117864	0.085809
25	JAMII BORA	2015	0.00%	0.00%	73.25%	0.00%	0.043665	0.012622	0.001057	0.005608	16781543	777949	10155694	7.224832	0.076602
25	JAMII BORA	2016	0.00%	0.00%	73.25%	0.00%	0.045884	-0.11912	-0.01065	-0.04663	15724254	2141177	9356471	7.19657	0.228845
25	JAMII BORA	2017	0.00%	0.00%	73.25%	0.00%	0.019085	-0.35059	-0.03834	-0.14262	12850795	2106308	8310978	7.10893	0.253437
25	JAMII BORA	2018	0.00%	0.00%	73.25%	0.00%	0.019038	-0.48267	-0.06039	-0.01896	11232453	2207231	8052384	7.050475	0.274109
25	JAMII BORA kindom bank	2019	0.00%	0.00%	73.25%	0.00%								#NUM!	#DIV/0!
25	JAMII BORA kingdom bank	2020	0.00%	0.00%	97.32%	0.00%								#NUM!	#DIV/0!

26	KCB	2009	17.00%	0.00%	24.80%	22.50%	0.07175	2.05	0.026411	0.203264	172381128	12172875	96557588	8.23649	0.126069
26	KCB	2010	17.00%	0.00%	24.80%	22.50%	0.07817	2.76	0.028562	0.18344	251316200	14583382	148113364	8.40022	0.098461
26	KCB	2011	17.00%	0.00%	24.80%	22.50%	0.070423	3.72	0.033209	0.246838	330663359	12228264	198724919	8.519386	0.061534
26	KCB	2012	17.53%	0.00%	24.80%	25.90%	0.083246	4.11	0.03316	0.224763	368018785	14750335	211664226	8.56587	0.069687
26	KCB	2013	17.53%	0.00%	24.80%	25.90%	0.084391	4.82	0.036693	0.226366	390851549	19227705	227721781	8.592012	0.084435
26	KCB	2014	17.53%	5.60%	26.99%	25.90%	0.073314	5.63	0.034362	0.22277	490338324	18404132	283732205	8.690496	0.064864
26	KCB	2015	17.53%	5.60%	26.99%	25.90%	0.070303	6.49	0.035161	0.241504	558094154	23477475	345968686	8.746707	0.06786
26	KCB	2016	17.53%	5.60%	26.99%	28.40%	0.079005	6.46	0.033134	0.204238	595239643	31812856	355745331	8.774692	0.089426
26	KCB	2017	17.53%	5.60%	26.99%	28.40%	0.074822	6.43	0.030472	0.185957	646668939	37496454	422684637	8.810682	0.08871
26	KCB	2018	17.53%	5.60%	26.99%	28.40%	0.06836	7.83	0.033592	0.21111	714312591	32691822	455880284	8.853888	0.071711
26	KCB	2019	17.53%	5.60%	26.99%	28.40%	0.080287	7.83	0.028006	0.193963	898572000		539747000	8.953553	0
26	KCB	2020	17.53%	5.60%	26.99%	28.40%	0.062182	6.1	0.019846	0.137646	987810000		595255000	8.994673	0
27	MIDDLE EAST BANK	2009	0.00%	0.00%	95.52%	9.78%	0.043085	2.486405	0.014871	0.06465	4236843	42785	1978537	6.627042	0.021625
27	MIDDLE EAST BANK	2010	0.00%	0.00%	95.52%	9.78%	0.043919	2.828933	0.015708	0.065809	4563759	61234	2347659	6.659323	0.026083
27	MIDDLE EAST BANK	2011	0.00%	0.00%	95.52%	9.78%	0.042061	3.72	0.020305	0.08562	4639160	64345	2564178	6.666439	0.025094
27	MIDDLE EAST BANK	2012	0.00%	0.00%	95.52%	9.78%	0.031418	1.75	0.007554	0.039442	5869715	74517	3144797	6.768617	0.023695
27	MIDDLE EAST BANK	2013	0.00%	0.00%	95.52%	9.78%	0.029279	1.049931	0.004452	0.020524	5976564	45623	2906785	6.776452	0.015695
27	MIDDLE EAST BANK	2014	0.00%	0.00%	95.52%	9.78%	0.037669	1.14885	0.005931	0.025711	4908234	47832	3349756	6.690925	0.014279
27	MIDDLE EAST BANK	2015	0.00%	0.00%	95.52%	9.78%	0.038409	2.762985	0.013733	0.059109	5098386	33421	2543869	6.707433	0.013138
27	MIDDLE EAST BANK	2016	0.00%	0.00%	95.52%	9.78%	0.041428	-2.62	-0.01267	-0.0556	5233522	1193400	3616626	6.718794	0.329976
27	MIDDLE EAST BANK	2017	0.00%	0.00%	95.52%	9.78%	0.034997	-0.99	-0.00492	-0.02168	5121036	1437922	2769120	6.709358	0.51927
27	MIDDLE EAST	2018	0.00%	0.00%	95.52%	9.78%	0.045496	0.1	0.000487	0.002255	5360864	1010272	2560398	6.729235	0.394576

	BANK														
27	MIDDLE EAST BANK	2019	0.00%	0.00%	95.52%	9.78%	0.039098	0.14	0.000427	0.003127	8466284	869702	5964756	6.927693	0.145807
27	MIDDLE EAST BANK	2020	0.00%	0.00%	95.52%	9.78%	0.030654	4.97	0.011428	0.098838	11021603	790359	7279704	7.042245	0.10857
28	NATIONAL BANK	2009	25.60%	15.60%	44.50%	0.00%	0.064827	4	0.02846	0.185004	51404408	1301757	13156455	7.711	0.098944
28	NATIONAL BANK	2010	25.60%	15.60%	44.50%	0.00%	0.072746	4.18	0.033684	0.203625	60026694	936290	20844636	7.778344	0.044918
28	NATIONAL BANK	2011	25.60%	15.60%	44.50%	0.00%	0.073999	3.19	0.022517	0.147862	68664516	1196826	28068218	7.836732	0.04264
28	NATIONAL BANK	2012	25.60%	15.60%	44.50%	0.00%	0.071101	1.49	0.010867	0.069833	67154805	2247476	28346668	7.827077	0.079285
28	NATIONAL BANK	2013	24.70%	15.60%	44.50%	0.00%	0.060957	2.24	0.011784	0.091988	92493034	4212274	39566678	7.966109	0.10646
28	NATIONAL BANK	2014	24.70%	20.00%	44.50%	0.00%	0.055263	2.86	0.006517	0.066097	122864886	7236684	65641491	8.089428	0.110246
28	NATIONAL BANK	2015	24.70%	20.00%	48.06%	0.00%	0.050931	-3.96	-0.00944	-0.10842	125295035	11762498	67803990	8.097934	0.173478
28	NATIONAL BANK	2016	22.50%	20.00%	48.06%	0.00%	0.06966	0.5	0.001281	0.01341	115114374	29987342	59339225	8.06113	0.505354
28	NATIONAL BANK	2017	22.50%	20.00%	48.06%	0.00%	0.061014	1.12	0.003456	0.053919	109942042	27657893	52361043	8.041164	0.528215
28	NATIONAL BANK	2018	22.50%	20.00%	48.06%	0.00%	0.052132	0.46	0.001356	0.022512	115143443	31461391	47778777	8.061239	0.65848
28	NATIONAL BANK	2019	22.50%	20.00%	48.06%	0.00%	0.056134	0	-0.00301	-0.02879	112028747	25175371	45871701	8.049329	0.548821
28	NATIONAL BANK	2020	0.00%	0.00%	100.00%	0.00%	0.028042	0	-0.0032	-0.03363	119225458	28659573	50273213	8.076369	0.570076
29	NIC BANK	2009	0.00%	2.30%	77.60%	0.00%	0.05076	2.75	0.022829	0.159846	47558241	1548270	32511082	7.677226	0.047623
29	NIC BANK	2010	0.00%	2.30%	77.60%	0.00%	0.054456	4.6	0.031584	0.223137	59013922	1570797	40754979	7.770954	0.038542
29	NIC BANK	2011	0.00%	2.30%	77.60%	0.00%	0.054182	5.54	0.034274	0.25726	78984005	1961277	56624621	7.897539	0.034636
29	NIC BANK	2012	0.00%	2.30%	77.60%	0.00%	0.050613	6.03	0.028028	0.196155	108348593	2332701	71540092	8.034823	0.032607
29	NIC BANK	2013	0.00%	2.30%	77.60%	0.00%	0.060035	6.71	0.026741	0.184263	121062739	4520926	83493313	8.08301	0.054147
29	NIC BANK	2014	0.00%	6.80%	76.80%	0.00%	0.054864	7.07	0.028239	0.176298	145780505	4032008	100575330	8.163699	0.040089
29	NIC BANK	2015	0.00%	6.80%	76.80%	0.00%	0.058765	7	0.027053	0.170238	165788268	13228534	114617644	8.219554	0.115414
29	NIC BANK	2016	0.00%	6.80%	76.80%	0.00%	0.071809	6.77	0.025554	0.142704	169458985	13587912	114666274	8.229065	0.1185
29	NIC BANK	2017	0.00%	6.80%	76.80%	0.00%	0.059578	5.93	0.020102	0.11938	206172460	14320960	118446485	8.314231	0.120907
29	NIC BANK	2018	0.00%	6.80%	76.80%	0.00%	0.056498	6.06	0.020289	0.11816	208407417	13456349	116853003	8.318913	0.115156
29	NIC/CBA	2019	0.00%	0.00%	53.31%	0.00%								#NUM!	#DIV/0!
29	NIC/CBA	2020	0.00%	0.00%	53.31%	0.00%								#NUM!	#DIV/0!

30	ORIENTAL BANK	2009	0.00%	8.60%	48.90%	0.00%	0.076627	0.738873	0.010685	0.006663	5689439	403892	2798327	6.755069	0.144333
30	ORIENTAL BANK	2010	0.00%	8.60%	48.90%	0.00%	0.090697	1.077276	0.015027	0.008952	5898461	349801	3030152	6.770739	0.11544
30	ORIENTAL BANK	2011	0.00%	8.60%	48.90%	0.00%	0.054088	0.774445	0.009961	0.061585	6396745	367103	3568435	6.805959	0.102875
30	ORIENTAL BANK	2012	0.00%	9.30%	48.90%	0.00%	0.078617	0.506334	0.006123	0.036727	6803457	390547	3900547	6.83273	0.100126
30	ORIENTAL BANK	2013	0.00%	9.30%	50.00%	0.00%	0.058617	0.552847	0.006117	0.036834	7435879	423905	4435856	6.871332	0.095563
30	ORIENTAL BANK	2014	0.00%	9.30%	50.00%	0.00%	0.049473	0.87	0.009156	0.049316	7857515	459000	4627523	6.895285	0.099189
30	ORIENTAL BANK	2015	0.00%	10.62%	50.00%	0.00%	0.04937	0.51	0.005049	0.019152	8496350	667022	5245063	6.929232	0.127171
30	ORIENTAL BANK	2016	0.00%	10.62%	50.00%	0.00%	0.065157	0.29	0.003396	0.011491	9920247	545199	6638054	6.996522	0.082132
30	ORIENTAL BANK	2017	0.00%	10.62%	50.00%	0.00%	0.053809	0.84	0.009125	0.031873	10576525	808524	7288835	7.024343	0.110926
30	ORIENTAL BANK	2018	0.00%	10.62%	50.00%	0.00%	0.049002	0.66	0.007841	0.026898	10515015	773122	7502022	7.02181	0.103055
30	ORIENTAL BANK	2019	0.00%	10.62%	50.00%	0.00%	0.030756		-0.00177	-0.00721	12393776	1410502	6811799	7.093204	0.207067
30	ORIENTAL BANK	2020	0.00%	10.62%	50.00%	0.00%	0.040904		0.002135	0.009027	12984554	1812217	7078318	7.113427	0.256024
31	PARAMOUNT BANK	2009	0.00%	89.60%	0.00%	6.00%	0.016635	66.32	0.007231	0.049898	6192344	684303	1873244	6.791855	0.365304
31	PARAMOUNT BANK	2010	0.00%	89.60%	0.00%	6.00%	0.017122	77.82	0.003726	0.028509	6893712	724376	2067412	6.838453	0.350378
31	PARAMOUNT BANK	2011	0.00%	89.60%	0.00%	6.00%	0.016816	88.45	0.003805	0.024816	7163274	798435	2453876	6.855112	0.325377
31	PARAMOUNT BANK	2012	0.00%	89.60%	0.00%	6.00%	0.017217	94.66	0.009285	0.059309	7254561	804295	2739612	6.860611	0.29358
31	PARAMOUNT BANK	2013	0.00%	89.60%	0.00%	6.00%	0.038664	110.25	0.010954	0.071479	8028877	946834	3572190	6.904655	0.265057
31	PARAMOUNT BANK	2014	0.00%	91.00%	0.00%	9.00%	0.032458	147.85	0.014213	0.10727	10402326	1062852	4447615	7.01713	0.238971
31	PARAMOUNT BANK	2015	0.00%	91.00%	0.00%	9.00%	0.052864	158.03	0.015013	0.102862	10525709	814626	5871717	7.022251	0.138737
31	PARAMOUNT BANK	2016	0.00%	91.00%	0.00%	9.00%	0.031383	106.29	0.011275	0.064644	9426931	778375	5799443	6.97437	0.134215
31	PARAMOUNT BANK	2017	0.00%	91.00%	0.00%	9.00%	0.039157	117.11	0.012275	0.066556	9541251	928435	5902031	6.979605	0.157308
31	PARAMOUNT	2018	0.00%	91.00%	0.00%	9.00%	0.036707	235.8	0.023846	0.139738	9887407	1069283	5642627	6.995082	0.189501

	BANK														
31	PARAMOUNT BANK	2019	0.00%	91.00%	0.00%	9.00%	0.03891		0.008709	0.051146	10443296	1262847	6462964	7.018838	0.195397
31	PARAMOUNT BANK	2020	0.00%	91.00%	0.00%	9.00%	0.038754		0.011696	0.069629	11378331	1345979	6827407	7.056079	0.197144
32	PRIME BANK	2009	0.00%	21.34%	74.50%	0.00%	0.037733	304.4	0.01705	0.131846	23699952	619381	10615380	7.374747	0.058348
32	PRIME BANK	2010	0.00%	21.34%	74.50%	0.00%	0.034724	340.9	0.018691	0.155568	32444424	607023	14836692	7.51114	0.040914
32	PRIME BANK	2011	0.00%	21.34%	74.50%	0.00%	0.046161	333.3	0.023716	0.222972	35184677	779113	18393706	7.546354	0.042358
32	PRIME BANK	2012	0.00%	21.34%	74.50%	0.00%	0.034756	304.2	0.021966	0.228664	43462888	775955	21150662	7.638119	0.036687
32	PRIME BANK	2013	0.00%	21.34%	74.50%	2.30%	0.050487	480	0.02913	0.247732	49460889	704349	26751542	7.694262	0.026329
32	PRIME BANK	2014	0.00%	21.34%	78.66%	2.30%	0.054114	578	0.031611	0.224436	54917674	665673	34481269	7.739712	0.019305
32	PRIME BANK	2015	0.00%	21.34%	78.66%	2.30%	0.051538	404.6	0.031125	0.231879	65001313	988594	41047741	7.812922	0.024084
32	PRIME BANK	2016	0.00%	21.34%	78.66%	2.30%	0.05435	380.8	0.029138	0.175738	65335455	1854804	39356307	7.815149	0.047129
32	PRIME BANK	2017	0.00%	21.34%	78.66%	2.30%	0.047473	368	0.024098	0.134265	76438199	2251548	38817386	7.88331	0.058004
32	PRIME BANK	2018	0.00%	21.34%	78.66%	2.30%	0.038182	304	0.020514	0.087735	98534455	2820997	36642825	7.993588	0.076986
32	PRIME BANK	2019	0.00%	21.34%	78.66%	2.30%	0.040347		0.022462	0.09992	108785527	4554770	36925060	8.036571	0.123352
32	PRIME BANK	2020	0.00%	21.34%	78.66%	2.30%	0.039992		0.018193	0.0849	116203591	4837979	35968295	8.06522	0.134507
33	SBM BANK	2009	0.00%	45.60%	55.60%	0.00%	0.042067	0.08	0.006128	0.069006	362477.2	2843.9	176523.8	5.559281	0.016111
33	SBM BANK	2010	0.00%	45.60%	55.60%	0.00%	0.044785	0.23	0.007679	0.133395	650948.5	10074.6	212046.9	5.813547	0.047511
33	SBM BANK	2011	0.00%	45.60%	55.60%	0.00%	0.046539	0.62	0.009658	0.176785	1030836	14091	409578	6.01319	0.034404
33	SBM BANK	2012	0.00%	45.60%	55.60%	0.00%	0.059994	1.68	0.020337	0.226098	1349922	33106	653724	6.130309	0.050642
33	SBM BANK	2013	0.00%	45.60%	55.60%	0.00%	0.069936	2.48	0.028888	0.31352	1691611	47855	827177	6.228301	0.057853
33	SBM BANK	2014	0.00%	45.60%	55.60%	0.00%	0.061848	3.57	0.026597	0.212341	3135003	40480	1661004	6.496238	0.024371
33	SBM BANK	2015	0.00%	45.60%	55.60%	0.00%	0.091131	5.76	0.035564	0.2841	4089082	90693	1489843	6.611626	0.060874
33	SBM BANK	2016	0.00%	0.00%	100.00%	100.00%	0.085341	0.36	0.002129	0.018077	4213460	381344	1352595	6.624639	0.281935
33	SBM BANK	2017	0.00%	0.00%	100.00%	100.00%	0.077105	3.79	0.017748	0.176231	5391850	338974	1066535	6.731738	0.317827
33	SBM BANK	2018	0.00%	0.00%	100.00%	100.00%	0.072657	6.73	0.024143	0.239985	7038258	132349	1462852	6.847465	0.090473
33	SBM BANK	2019	0.00%	0.00%	100.00%	100.00%	0.038263		0.012464	0.114746	72519356	14980134	15463645	7.860454	0.968732
33	SBM BANK	2020	0.00%	0.00%	100.00%	100.00%	0.047144		0.008267	0.073797	79189802	16225120	25023214	7.898669	0.648403

34	SIDIAN BANK	2009	0.00%	0.00%	98.00%	0.00%	0.106458	42.89265	0.013345	0.091006	7325658	980945	4800936	6.864847	0.204324
34	SIDIAN BANK	2010	0.00%	0.00%	98.00%	0.00%	0.113954	22.2	0.006602	0.043747	7670049	1236539	5252438	6.884798	0.235422
34	SIDIAN BANK	2011	0.00%	0.00%	98.00%	0.00%	0.11768	76.1	0.018604	0.130253	9318715	900874	6754243	6.969356	0.133379
34	SIDIAN BANK	2012	0.00%	0.00%	98.00%	0.00%	0.13727	86.03	0.02054	0.128393	9546050	987660	6954783	6.979824	0.142012
34	SIDIAN BANK	2013	0.00%	0.00%	98.00%	0.00%	0.107541	157.91	0.027684	0.192718	13000918	72130	8693764	7.113974	0.008297
34	SIDIAN BANK	2014	0.00%	2.00%	98.00%	0.00%	0.105082	225.53	0.032531	0.211386	15801439	776423	10453714	7.198697	0.074272
34	SIDIAN BANK	2015	0.00%	2.00%	98.00%	0.00%	0.086718	126.71	0.019487	0.097022	19106557	1607630	12519387	7.281182	0.128411
34	SIDIAN BANK	2016	0.00%	2.00%	98.00%	0.00%	0.091049	9.54	0.001344	0.00725	20875499	2458729	13571012	7.319637	0.181175
34	SIDIAN BANK	2017	0.00%	2.00%	98.00%	0.00%	0.054261	143.46	0.021853	0.122371	19301752	2595562	11409325	7.285597	0.227495
34	SIDIAN BANK	2018	0.00%	2.00%	98.00%	0.00%	0.041967	96.62	0.01561	0.09794	25329169	2941718	13134315	7.403621	0.223972
34	SIDIAN BANK	2019	0.00%	2.00%	98.00%	0.00%	0.035306		0.00338	0.021913	26451638	3258027	14526066	7.422453	0.224288
34	SIDIAN BANK	2020	0.00%	2.00%	98.00%	0.00%	0.025631		0.001864		33499603	2327489	18997464	7.52504	0.122516
35	SPIRE BANK	2009	0.00%	100.00%	0.00%	0.00%	0.029275	6.17	-0.1269	115.4228	4491375	982325	1953299	6.652379	0.502906
35	SPIRE BANK	2010	0.00%	100.00%	0.00%	0.00%	0.028457	0.5	-0.01027	-0.11784	10398805	1198108	4851414	7.016983	0.246961
35	SPIRE BANK	2011	0.00%	100.00%	0.00%	0.00%	0.030294	-0.5	0.005596	0.060071	12926902	589038	6635194	7.111494	0.088775
35	SPIRE BANK	2012	0.00%	100.00%	0.00%	0.00%	0.02432	-1.4	-0.03416	-0.66722	14108996	805083	7538422	7.149496	0.106797
35	SPIRE BANK	2013	0.00%	100.00%	0.00%	0.00%	0.053237	0.13	0.00357	0.040511	15562476	1371225	9029000	7.192079	0.151869
35	SPIRE BANK	2014	0.00%	100.00%	0.00%	0.00%	0.055414	-0.69	-0.01968	-0.28264	16589359	3027971	10067792	7.21983	0.300758
35	SPIRE BANK	2015	0.00%	49.00%	51.00%	0.00%	0.042327	-0.78	-0.03361	-0.2351	14469562	3387828	8321620	7.160455	0.407112
35	SPIRE BANK	2016	0.00%	49.00%	51.00%	0.00%	0.034331	-0.92	-0.05446	-0.41361	13802498	1322155	7433605	7.139958	0.177862
35	SPIRE BANK	2017	0.00%	49.00%	51.00%	0.00%	0.025486	-1.17	-0.10101	-0.94804	11147949	2349262	5238814	7.047195	0.448434
35	SPIRE BANK	2018	0.00%	49.00%	51.00%	0.00%	0.006618	-2.34	-0.24449	2.18934	9223078	2686049	4445622	6.964876	0.604201
35	SPIRE BANK	2019	0.00%	49.00%	75.00%	0.00%	0.010102		-0.06881	0.855155	6860301	2631593	3311899	6.836343	0.794587
35	SPIRE BANK	2020	0.00%	49.00%	75.00%	0.00%	-0.0213		-0.24793	6.967251	5114288	2711017	2556373	6.708785	1.060494
36	STANDARD CHARTERED	2009	0.00%	0.00%	85.00%	73.89%	0.057655	16.44	0.035331	0.340821	133909119	1473750	56694876	8.12681	0.025994
36	STANDARD CHARTERED	2010	0.00%	0.00%	85.00%	73.89%	0.058644	18.55	0.030558	0.216036	142880029	1205556	60336829	8.154972	0.01998
36	STANDARD	2011	0.00%	0.00%	85.00%	73.89%	0.060002	19.75	0.035551	0.28374	164181638	1030827	2542427	8.215325	0.40545

	CHARTERED														
36	STANDARD CHARTERED	2012	0.00%	2.40%	85.00%	73.89%	0.072719	26.51	0.041143	0.262825	195492999	2180974	112694523	8.291131	0.019353
36	STANDARD CHARTERED	2013	0.00%	2.40%	85.00%	73.89%	0.0759	29.33	0.041883	0.256349	220523869	3848116	129672004	8.343456	0.029676
36	STANDARD CHARTERED	2014	0.00%	2.40%	85.00%	73.89%	0.08031	33.11	0.046732	0.257214	222635993	10752493	122749233	8.347595	0.087597
36	STANDARD CHARTERED	2015	0.00%	2.40%	85.00%	73.89%	0.07724	17.6	0.026538	0.151864	234130556	14697920	115125427	8.369458	0.127669
36	STANDARD CHARTERED	2016	0.00%	2.40%	85.00%	73.89%	0.077318	24.8	0.034709	0.197854	250274108	15038172	122711038	8.398416	0.122549
36	STANDARD CHARTERED	2017	0.00%	2.40%	85.00%	73.89%	0.064833	18.5	0.022877	0.146301	285124538	17620531	126294470	8.455035	0.139519
36	STANDARD CHARTERED	2018	0.00%	2.40%	85.00%	73.89%	0.067761	22.44	0.027662	0.173704	284691002	21660851	118651550	8.454374	0.182559
36	STANDARD CHARTERED	2019	0.00%	2.40%	85.00%	73.89%	0.063982	23.35	0.029738	0.19037	302295903	20057683	128690341	8.480432	0.15586
36	STANDARD CHARTERED	2020	0.00%	2.40%	85.00%	73.89%	0.05857	13.66	0.016355	0.106126	325872857	22337323	121524227	8.513048	0.18381
37	TRANSNATIONAL BANK	2009	0.00%	3.80%	91.80%	0.00%	0.099386	0.77	0.026797	0.06802	3364458	504296	1688664	6.526915	0.298636
37	TRANSNATIONAL BANK	2010	0.00%	3.80%	91.80%	0.00%	0.073026	1.22	0.029892	0.092386	4761852	536624	1937580	6.677776	0.276956
37	TRANSNATIONAL BANK	2011	0.00%	3.80%	91.80%	0.00%	0.073128	1.65	0.027801	0.116204	7286906	380716	3308068	6.862543	0.115087
37	TRANSNATIONAL BANK	2012	0.00%	3.80%	91.80%	0.00%	0.062687	1.74	0.024245	0.116366	8801382	512061	4238908	6.944551	0.1208
37	TRANSNATIONAL BANK	2013	0.00%	3.80%	95.20%	0.00%	0.072324	0.79	0.016372	0.084598	9657867	672524	5144709	6.984881	0.130721
37	TRANSNATIONAL BANK	2014	0.00%	4.80%	95.20%	0.00%	0.07594	0.63	0.012277	0.065654	10239922	529263	6009247	7.010297	0.088075
37	TRANSNATIONAL	2015	0.00%	4.80%	95.20%	0.00%	0.082385	0.84	0.016075	0.08266	10452691	733408	6649506	7.019228	0.110295

	BANK														
37	TRANSNATIONAL BANK	2016	0.00%	4.80%	95.20%	0.00%	0.083709	0.55	0.010521	0.052418	10372441	736146	6367429	7.015881	0.115611
37	TRANSNATIONAL BANK	2017	0.00%	4.80%	95.20%	0.00%	0.067069	0.18	0.003557	0.01753	10241368	1595247	6604120	7.010358	0.241553
37	TRANSNATIONAL BANK	2018	0.00%	4.80%	95.20%	0.00%	0.055506	0.36	0.007019	0.037251	10235524	1786382	6625522	7.01011	0.269621
38	UBA BANK	2009	0.00%	0.00%	100.00%	100.00%	0.010374	-0.17299	-0.172	-0.30791	1798358	17932	321874	6.254876	0.055711
38	UBA BANK	2010	0.00%	0.00%	100.00%	100.00%	0.010417	-0.16655	-0.1561	-0.27114	1907864	33023	389043	6.280547	0.084883
38	UBA BANK	2011	0.00%	0.00%	100.00%	100.00%	0.007947	-0.16299	-0.11263	-0.24504	2587634	42987	429768	6.412903	0.100024
38	UBA BANK	2012	0.00%	0.00%	100.00%	100.00%	0.007539	-0.16073	-0.09829	-0.23579	2923811	53617	439993	6.465949	0.121859
38	UBA BANK	2013	0.00%	0.00%	100.00%	100.00%	0.032778	-0.21112	-0.07335	-0.25692	3709628	15633	789933	6.56933	0.01979
38	UBA BANK	2014	0.00%	0.00%	100.00%	100.00%	0.013602	-0.18028	-0.0593	-0.24763	4755787	52343	734061	6.677222	0.071306
38	UBA BANK	2015	0.00%	0.00%	100.00%	100.00%	0.0142	-0.20817	-0.03375	-0.2347	7781236	57839	2733280	6.891049	0.021161
38	UBA BANK	2016	0.00%	0.00%	100.00%	100.00%	0.039397	0.019258	0.004338	0.011336	5601281	68743	3058201	6.748287	0.022478
38	UBA BANK	2017	0.00%	0.00%	100.00%	100.00%	0.048491	0.014749	0.002861	0.008607	6504732	151711	3270289	6.813229	0.046391
38	UBA BANK	2018	0.00%	0.00%	100.00%	100.00%	0.030673	0.042056	0.003461	0.024406	15332118	441721	3447577	7.185602	0.128125
38	UBA BANK	2019	0.00%	0.00%	100.00%	100.00%	0.046583	0.053568	0.004201	0.030149	16088319	882862	3629616	7.206511	0.243238
38	UBA BANK	2020	0.00%	0.00%	100.00%	100.00%	0.029027	0.19917	0.011524	0.100797	21806135	818316	3304801	7.338579	0.247614
39	VICTORIA COMMECIAL BANK	2009	0.00%	23.60%	36.50%	26.34%	0.05527	10.00924	0.033985	0.194952	5876304	0	2540987	6.769104	0
39	VICTORIA COMMECIAL BANK	2010	0.00%	23.60%	36.50%	26.34%	0.059642	10.8	0.034559	0.194759	6215400	0	2962241	6.793469	0
39	VICTORIA COMMECIAL BANK	2011	0.00%	23.60%	36.50%	26.34%	0.061588	11.54	0.030117	0.183835	7645235	0	4110436	6.883391	0
39	VICTORIA COMMECIAL BANK	2012	0.00%	23.60%	36.50%	26.34%	0.063915	11.56	0.033957	0.172166	10322819	0	5291220	7.013798	0

39	VICTORIA COMMECIAL BANK	2013	0.00%	23.60%	36.50%	26.34%	0.056517	13.42	0.031655	0.170838	13644242	0	8363452	7.134949	0
39	VICTORIA COMMECIAL BANK	2014	0.00%	23.60%	36.50%	26.34%	0.051863	14.36	0.026928	0.161481	17244092	0	10979238	7.23664	0
39	VICTORIA COMMECIAL BANK	2015	0.00%	26.50%	38.02%	26.34%	0.056069	21.69	0.035654	0.203274	20020072	0	13124420	7.301466	0
39	VICTORIA COMMECIAL BANK	2016	0.00%	26.50%	38.02%	26.34%	0.05124	18.6	0.026442	0.117073	22403481	0	15292829	7.350316	0
39	VICTORIA COMMECIAL BANK	2017	0.00%	26.50%	38.02%	26.34%	0.049425	14.72	0.023751	0.109977	25985160	16855	18870101	7.414725	0.000893
39	VICTORIA COMMECIAL BANK	2018	0.00%	26.50%	38.02%	26.34%	0.039981	10.42	0.013514	0.073287	32336955	696424	22586671	7.509699	0.030833
39	VICTORIA COMMECIAL BANK	2019	0.00%	26.50%	38.02%	26.34%	0.044841		0.014614	0.08293	36072410	1204434	23789164	7.557175	0.05063
39	VICTORIA COMMECIAL BANK	2020	0.00%	26.50%	38.02%	26.34%	0.029548		0.00942	0.051634	37224447	1567467	23963576	7.570828	0.06541
