# Supplementary material for: Ownership structure and financial performance: Evidence from Kenyan commercial banks
Source: PLoS One. 2022 May 20;17(5):e0268301. doi: 10.1371/journal.pone.0268301 (PMC9122193; doi:10.1371/journal.pone.0268301)
Supplement: Attachment — Submitted filename: RESPONSE TO REVIEWERS.docx [file pone.0268301.s002.docx]

**TO THE EDITOR, PLOS ONE**

This letter shows how recommendation by the editors and reviewer were addressed. The correction matrix below shows how each of the recommendation was addressed in the revised manuscript.

**CORRECTION MATRIX**

| **COMMENTS** | **ACTION TAKEN/CORRECTION DONE** | **CONTENT/PAGE AFFECTED** |
| --- | --- | --- |
| Please include the following items when submitting your revised manuscript:   - A rebuttal letter that responds to each point raised by the academic editor and reviewer(s). You should upload this letter as a separate file labeled 'Response to Reviewers'. - A marked-up copy of your manuscript that highlights changes made to the original version. You should upload this as a separate file labeled 'Revised Manuscript with Track Changes'. - An unmarked version of your revised paper without tracked changes. You should upload this as a separate file labeled 'Manuscript'. | Rebuttal letter attached  Marked up copy highlighting the changes (highlighted in yellow or colored red) was uploaded.  Unmarked revised version/copy uploaded without tracked changes was uploaded. |  |
| When submitting your revision, we need you to address these additional requirements.  1. Please ensure that your manuscript meets PLOS ONE's style requirements, including those for file naming | The whole paper was aligned to meet PLOS ONE style requirement | Whole paper |
| Please review your reference list to ensure that it is complete and correct. If you have cited papers that have been retracted, please include the rationale for doing so in the manuscript text, or remove these references and replace them with relevant current references. Any changes to the reference list should be mentioned in the rebuttal letter that accompanies your revised manuscript. If you need to cite a retracted article, indicate the article’s retracted status in the References list and also include a citation and full reference for the retraction notice. | The references were reviewed and aligned to PLOS One Style requirements. | Reference section |
| REVIEWERS COMMENTS:  Reviewer #1: It was understood that a lot of effort was spent on this study. The topic is current. Literature research is sufficient. English level is appropriate. Although the number of pages is high, it is appropriate when the whole study is taken into account. However, some mistakes in the citations should be corrected. For example, the reference to Kenya's central bank on page 7 is dated 2016. It is not in this date in bibliography. On page 8, "Amneh Amneh, Hussam & Mahmoud, 2021" also missed in the references. . In addition, in some citations the authors are written individually, in some of them "et al." used. One format of citations should be preferred. | All in-text citations and references were rectified as per the PLOS one requirements. | Whole document |
